# Developing a More Sustainable Protein and Amino Acid Supply of Laying Hens in a Split Feeding System

**DOI:** 10.3390/ani14203006

**Published:** 2024-10-17

**Authors:** Boglárka Horváth, Patrik Strifler, Nikoletta Such, László Wágner, Károly Dublecz, Henrik Baranyay, László Bustyaházai, László Pál

**Affiliations:** 1 Institute of Physiology and Nutrition, Hungarian University of Agriculture and Life Sciences, Georgikon Campus, 8360 Keszthely, Hungary; boglarka.horvath@ubm.hu (B.H.); striflerpatrik@gmail.com (P.S.); such.nikoletta.amanda@uni-mate.hu (N.S.); wagner.laszlo@uni-mate.hu (L.W.); dublecz.karoly@uni-mate.hu (K.D.); 2 UBM Feed Company, 2085 Pilisvörösvár, Hungary; henrik.baranyay@ubm.hu (H.B.); laszlo.bustyahazai@ubm.hu (L.B.)

**Keywords:** laying hen, split feeding, low protein, nitrogen emission, sustainability

## Abstract

**Simple Summary:**

In contrast to a conventional layer diet with the same nutrient content during the day, morning and afternoon feeds with different compositions are fed in a split feeding system. This system is based on the sequential need for energy, amino acids, and calcium during egg formation and might be suitable to decrease the intake and emission of nitrogen and develop a more sustainable nutrition plan for laying hens. Therefore, our study investigated the reduced or low crude protein and adjusted amino acid supply of laying hens in a split feeding system. The investigated split system resulted in a similar egg production with reduced protein intake, a more favorable feed conversion ratio, increased eggshell thickness, better digestibility of some amino acids, and a decreased nitrogen emission compared to conventional feeding. The decrease in the crude protein content of both morning and afternoon diets by 2% while maintaining important essential amino acid levels constantly led to a decreased protein intake, higher laying rate, lower egg weight, and similar nitrogen emissions of laying hens.

**Abstract:**

Two model experiments were conducted to investigate the different protein and amino acid supply of laying hens fed split feeding (SF) diets. In Experiment 1, one conventional (C) and one SF dietary treatment were established, and the diets were implemented for 12 weeks. The concentrations of crude protein, SID Lys, Met, Met + Cys Arg, Val, Thr, Leu, Ile, and Trp of the SF morning diet were the same as in the C diet. The crude protein content of the SF afternoon diet was lower (92%), while the SID values of Lys, Met, Met + Cys were identical compared to the C diet (100%). The SF treatment resulted in a reduced protein/N intake, better feed conversion ratio, higher eggshell thickness and apparent ileal digestibility of Asp, Leu, Lys, Gly, and Ser, and lower concentration of N forms (total, fecal, NH_4_^+^, uric acid, urinary) in the excreta compared to the C treatment. In Experiment 2, a control and a low protein (−2% crude protein but the same SID values of Lys, Met, Met + Cys, Thr, Val) SF treatment were compared for 6 weeks. The low protein SF treatment led to a decreased protein/N intake, higher laying rate, lower egg weight, higher ileal digestibility of Ala, Asp, Leu, and Ile, and similar N emission compared to the control SF treatment.

## 1. Introduction

Feeding a complete mash feed to laying hens with the same nutrient content during the day is the conventional and widely used approach. However, alternative systems, like sequential or split, free choice, and loose-mix feeding, can follow the hourly changing nutrient demand of the daily egg formation cycle more precisely [1]. These concepts can be used to prevent the oversupply of nutrients and to increase sustainability and production efficiency [1,2,3]. In a sequential or split feeding (SF) system, morning and afternoon feeds with different compositions are distributed in the same trough and may be the most suitable alternative system for large-scale layer farms [1]. The most accepted SF system is based on the sequential need for energy, amino acids (AAs), and calcium (Ca) during the ovulation–oviposition cycle [2,3,4]. In theory, the requirement for essential AAs is higher immediately after oviposition, in the first half of the day, to synthesize albumen and shell membrane proteins [4]. Thereafter, the intense shell formation during the afternoon and evening period increases the Ca requirement [5]. Based on this, a high energy, high protein, and low Ca diet is fed in the morning, while a low energy, low protein, and high Ca diet is offered in the afternoon [1,6,7]. The other concept of the SF system combines a high energy–low protein diet in the morning and a high protein–high Ca mixture in the afternoon [8,9,10,11,12]. The high energy–low protein morning diet is usually whole wheat or another grain. A biological explanation of the high protein content of afternoon diets is lacking but it might be justified as a compensation for the low protein content of the whole grain and to ensure a satisfying daily total protein intake.

A lot of trials with the SF system demonstrated a similar egg production with lower feed and protein/nitrogen (N) intake and a better feed conversion ratio compared to a conventional diet [2,6,8,9]. Using 16% crude protein content in the morning and 10% crude protein in the afternoon diet resulted in a lower protein intake and better protein retention but lower egg production than the control crude protein level of 16% both in the morning and afternoon diets (16–16%) [10]. So, the SF concept might be suitable to decrease nitrogen (N) emissions and increase the sustainability of laying hen nutrition. Low protein (LP) diets with adjusted digestible AA levels in conventional feeding can be applied to reduce the N excretion of laying hens without adverse effects on laying performance and egg quality. The decrease in dietary crude protein levels from 17 to 13.5% [11] or from 16 to 13% [12] with increased crystalline AA supplementation could decrease N excretion by 35 or 45%, respectively, and maintain egg production, egg weight, and quality. However, the 13% or lower crude protein level without a sufficient AA supply may decrease egg weight [13,14,15].

The LP concept in the SF system has not been investigated yet. However, the afternoon diet in a SF system without whole grain feeding can already be considered as a LP diet compared to the morning SF or a conventional diet. The advisable crude protein levels of morning and afternoon LP diets and the adjusted digestible (Standardized Ileal Digestible, SID) AA concentrations require further studies. A detailed complex investigation evaluating the N balance (N intake, emission, and retention), N forms of excreta, and ileal AA digestibility in the SF system has also been lacking. Furthermore, the different composition of morning and afternoon diets can decrease the Ca and P intake of hens so the SF system may decrease the Ca and P emissions as well [3]. Therefore, two model experiments were conducted in our study to investigate the different protein and AA supplies of laying hens fed SF diets. The ‘ideal protein concept’ or ‘ideal amino acid profile’ on a digestible (SID) AA basis has been applied as a new approach in the SF system investigation. Experiment 1 (Exp. 1) used a conventional and an SF system, while in Experiment 2 (Exp. 2), normal protein SF and low protein SF diets were compared with the measurements of performance parameters, egg quality, retention, and emissions of N, Ca, P and apparent ileal AA digestibility.

## 2. Materials and Methods

### 2.1. Experimental Animals and Housing

The two model trials were carried out at the experimental farm of the Institute of Physiology and Nutrition, Georgikon Campus, Hungarian University of Agriculture and Life Sciences (Keszthely, Hungary). A total of 48 Brown Nick (28 weeks old) and 48 Hy-Line Brown (47 weeks old) laying hens were obtained from a commercial production farm (Fuchs Tojás Ltd., Kolontár, Hungary) in Exp. 1 and 2, respectively. Hens were housed individually in wire cages (1056 cm^2^ surface area) equipped with a nipple drinker and feeding trough in a 2-tier system. Within a tier, 6 hens housed next to each other belonged to different treatments and this block of 6 hens was repeated 8 times. After the hens arrived at the experimental farm, a one-week-long adaptation period preceded the experiment when they received the same feed as in the production farm. The room temperature was set to be 20 ± 0.6 °C and the hens received 16 h of light and 8 h of dark per day with 30 lux light intensity in both experiments.

### 2.2. Experimental Treatments

In Exp. 1, one conventional (control, C) and one split feeding (SF) dietary treatment with 24 replicates of individually housed hens in each were established. The composition and nutrient contents of the experimental diets are shown in Table 1. The experimental diets were fed for 12 weeks, from 29 to 41 weeks of age. A total of 120 g feed/hen was provided daily in two portions in both feeding systems. This quantity represented 104% of the average theoretical requirement of 115 g/bird and could be considered as ad libitum supply. In the morning (at 8:00), 40% of the total daily feed amount was fed when the lights were switched on and was available for 7 h after lights on. The remaining 60% of the feed was distributed in the afternoon (at 15:00) and was available for 9 h until lights off. The feed left over from the previous distribution was always removed before the next distribution. The control diet was formulated in line with the breeder’s recommendations (Brown Nick Management Guide, 2024; H&N International) and the hens consumed the same diet during the day. The morning diet of the SF treatment had a higher energy (AMEn; 102.7%) and lower Ca content (80.4%) than the C diet, while the SF afternoon diet contained a lower energy level (96.4%) and higher Ca concentration (113.4%) compared to the C diet (100%). The crude protein content of the SF morning diet was set to be the same as the control (100%) and the crude protein content of the SF afternoon diet (92.0%) was lower compared to the C diet. The concentrations of SID lysine (Lys), methionine (Met), and methionine plus cystine (Met + Cys) in the SF morning and afternoon diets, as well as the SID values of other main essential AAs (arginine (Arg), valine (Val), threonine (Thr), leucine (Leu), isoleucine (Ile), tryptophan (Trp)) in the SF morning diet, were nearly the same as in the C diet. The SID values of Arg, Val, Thr, Leu, Ile, and Trp in the SF afternoon diet were nearly as low as the crude protein content compared to the C diet (approximately 90%). The ratio of coarse and fine limestone was the same (70:30%) in the C and SF diets.

In Exp. 2, two SF systems, a control (SF-C) and a low protein (SF-LP) treatment, were compared. The composition and nutrient contents of experimental diets are shown in Table 2. Experimental diets were fed for 6 weeks, from 47 to 53 weeks of age. The daily feed quantity and feed presentation schedule were the same as in Exp. 1. The energy and Ca contents of the morning and afternoon diets of the two split systems were identical and showed similar differences compared to the theoretical conventional diet of the breed (Hy-Line Performance Guide, 2024; Hy-Line International) as described for Exp. 1. The crude protein and essential SID AA levels of the SF-C morning diet were formulated in line with the breeder’s recommendations (Hy-Line Performance Guide, 2024; Hy-Line International). The SF-LP morning and afternoon diets contained 2.0% less crude protein than the corresponding morning and afternoon SF-C diets. However, the concentrations of SID Lys, Met, Met + Cys, Thr, and Val were not changed accordingly; they were the same in the morning SF-C and SF-LP diets and in the afternoon SF-C and SF-LP diets. Crystalline Arg, Leu, Ile, and Trp were not used to adjust the SID level of these essential AAs, and their SID level was decreased with the reduction in the crude protein content of SF-LP diets compared to the SF-C diets. The relative reduction in the dietary crude protein content was nearly 13–14% and the relative decrease in the SID Arg, Leu, Ile, and Trp levels was approximately 20% in the SF-LP diets compared to the SF-C diets.

### 2.3. Data Collection and Measurements

In both experiments, the individual body weight (BW) of hens was measured weekly. The morning and afternoon feed intake (FI) of birds, laying percentage, and egg weight were recorded daily. At the end of the experiment, the following parameters were calculated per hen for the whole trial period: average daily FI, average egg weight, total and daily egg mass, and feed conversion ratio (FCR). On the last days of the experiments, one egg per hen was collected for the measurements of egg quality parameters. The collected eggs were refrigerated, being stored at 4 °C. After 24 h, the eggs were settled at room temperature for 30 min, and the egg quality parameters were measured with a Digital Egg Tester 6500 (Nabel Co., Kyoto 601-8444 Japan).

During the last week of both experiments, the hens consumed diets supplemented with 0.5% titanium dioxide as an indigestible internal marker. After a four-day adaptation period, representative samples from the homogenized daily excreta were collected from each bird, for two consecutive days. The two representative samples per bird were pooled, mixed thoroughly, frozen, and stored at −20 °C until the analyses. At 8:00 on the last day of both experiments, birds without morning feed intake were euthanized by CO_2_ exposure and ileal digesta samples of each bird were collected. The section of the small intestine between Meckel’s diverticulum and the site 2 cm anterior to the ileocecal–colonic junction was removed. Digesta samples were obtained by flushing the terminal two-thirds of the removed section of the small intestine with deionized water [16]. Digesta samples of three (Exp. 1) or two (Exp. 2) hens were pooled and 8 (Exp. 1) or 12 (Exp. 2) pooled samples per treatment group were frozen and stored at −20 °C until the analyses.

Excreta and digesta samples were dried in a forced-air oven at 60 °C for 72 h before the analyses. Diets, dried ileal digesta, and excreta samples were ground using a grinding mill (Retsch ZM 100, Retsch GmbH and Co., K.G., Haan, Germany). All analyses were performed in duplicate. Dry matter content was determined by drying samples at 105 °C for 24 h in an exicator. The total nitrogen content of the diets, excreta, and digesta samples was determined according to the Kjeldahl method with a Foss-Kjeltec 8400 Analyzer Unit (Nils Foss Allé 1, DK-3400 Hilleroed, Denmark). The concentration of ammonium–N in the excreta was measured by the method of Peters [17] and the uric acid–N was measured as described by Marquardt [18]. All N parameters were adjusted to the dry matter basis. The sum of ammonium–N and uric acid–N was considered as urinary N content [19].

Diet and excreta samples were prepared by the dry ash method (AOAC Official Method 927.02) before the concentration of calcium in the supernatant was measured using flame atomic absorption spectrometry (Perkin Elmer AAnalyst 200, PerkinElmer, Inc., Wellesley, MA, USA). The phosphorus concentration of the samples was measured by the photometric method (AOAC Official Method 965.17) using a Biochrom Libra S12 UV-VIS spectrophotometer (Biochrom Ltd. Cambridge, UK). The concentration of AAs in the diet and digesta samples was analyzed after oxidation in a mixture of hydrogen peroxide and phenolic formic acid solution and hydrolyzation in a 6 M HCl solution (procedure ISO 13903:2005). Separation and detection of AAs were carried out using an automatic AA analyzer unit (Amino Acid Analyser, AAA 400; Ingos, Czech Republic). The TiO_2_ concentration of the experimental diets, excreta, and digesta samples was determined using a UV–spectroscopy assay [20].

### 2.4. Calculations and Statistical Analysis

The total tract nitrogen, calcium, and phosphorus retention were calculated using the following equation [21]: Apparent retention = 1 − [([TiO_2_] diet/[TiO_2_] excreta) × ([Item] excreta/[Item] diet)], where TiO_2_ diet, TiO_2_ excreta, Item diet, and Item excreta are the concentrations of the marker and the nitrogen, calcium, and phosphorus in the diet and in the excreta samples. The apparent ileal digestibility coefficients of AAs were calculated according to the equation for retention, but the concentrations of TiO_2_ and AA in the digesta were used. The average dietary concentrations of the marker, macro elements, and AAs in the split feeding diets were calculated with the consideration of the daily ration of morning and afternoon diets as follows: Average concentration = (concentration in the morning diet × 0.4) + (concentration in the afternoon diet × 0.6).

The averages of the examined parameters were analyzed as a completely randomized design by an independent samples *t*-test with dietary treatments as the main effects after testing the homogeneity of variances (Levene-test). The t-statistic is calculated in the following way: t = (x_1_ − x_2_)/√[(s_1_^2^/n_1_) + (s_2_^2^/n_2_)], where x_1_ and x_2_ are sample means, s_1_^2^ and s_2_^2^ are sample variances, and n_1_ and n_2_ are sample sizes. All statistical analyses were carried out by the software package SPSS 29.0 for Windows (IBM Corp., Armonk, NY, USA). Statistical significance has been declared at *p* < 0.05.

## 3. Results

### 3.1. Body Weight and Performance Parameters

The dietary treatments did not influence the BW of hens during both trials (*p* > 0.05). The BW of hens at the end of Exp. 1 were 1973 ± 26 g vs. 1943 ± 19 g in the C vs. SF groups, while the BW of hens were 1861 ± 29 g vs. 1852 ± 24 g in the SF-C vs. SF-LP groups at the end of Exp. 2. The effects of dietary treatments on feed and protein intake are shown in Table 3, while the Ca and P intake of experimental animals can be seen in Table 4. In Exp. 1, FI and protein intake in total and the morning and afternoon phases as well were significantly lower in the SF group than in the C group. Similarly, the SF treatment decreased the Ca and P intake of hens in the morning and afternoon phases as well as the total daily intake of these elements. The dietary treatments in Exp. 2 did not significantly affect the FI and Ca intake of hens. However, the feeding of SF-LP diets decreased the morning, afternoon, and total protein and P intake of hens compared to the SF-C diets. The parameters of laying performance obtained in the trial are shown in Table 5. In Exp. 1, the SF treatment resulted in a lower FI and a more favorable FCR of hens compared to the C treatment (*p* < 0.05). However, egg production, egg weight, and egg mass were not significantly affected by experimental feeding. In Exp. 2, the feeding of SF-LP diets led to a higher laying rate and lower egg weight compared to the SF-C diets, while the egg mass, FI, and FCR were similar in both groups.

### 3.2. Egg Quality Parameters

The eggshell thickness was higher in the SF treatment group than in the C group in Exp. 1 (Table 6). In Exp. 2, the egg weight results during this measurement represented the average egg weight calculated for the whole study (Table 5). The feeding of SF-LP diets decreased the egg weight compared to the feeding of SF-C diets. However, the albumen height and Haugh unit were significantly higher in the SF-LP group than in the case of SF-C animals.

### 3.3. Nitrogen Intake, Retention, and Emission

The N intake, retention, and emission of experimental animals is shown in Table 7. The hens in the split-feeding group consumed a lower amount of N daily (*p* < 0.001) and their N emission was also significantly lower (*p* < 0.05) than the hens in the conventional group in Exp. 1.

The decreased crude protein content of SF morning and afternoon diets in Exp. 2 resulted in a lower N intake of hens (*p* < 0.001) but did not affect the emitted amount of N significantly compared to the SF-C treatment. The N retention of birds was not influenced by dietary treatments in either experiment (*p* > 0.05).

### 3.4. Concentration of Dry Matter and Nitrogen Forms in the Excreta

The concentration of dry matter (DM) and the different forms of N are presented in Table 8. In Exp. 1, the SF treatment decreased all the measured forms of N (fecal, NH_4_^+^, uric acid, urinary) and total N content of excreta in a significant manner. In Exp. 2, the SF-LP treatment increased the DM content of excreta compared to the SF-C treatment. The effect of feeding low protein diets on the measured N forms was not significant.

### 3.5. Apparent Ileal Digestibility of Amino Acids

In Exp. 1, the significantly higher ileal digestibility of Asp, Leu, Lys, Gly, and Ser was measured with layers in the split feeding system compared to the hens in the conventional feeding system (Table 9). The SF-LP diets resulted in higher digestibility of Ala, Asp, Leu, and Ile in comparison with the SF-C treatment in Exp. 2.

### 3.6. Intake, Retention, Emission, and Excreta Concentration of Calcium and Phosphorus

The intake, retention, emission, and excreta concentration of Ca and P can be found in Table 10 and Table 11, respectively. The retention of Ca was higher, while the intake, emission, and excreta concentration of Ca was significantly lower in the SF than in the C treatment group in Exp. 1. Although the P intake was significantly higher in the C group than in the SF group, the difference observed in the P retention results increased the excreta P concentration in the SF compared to the C treatment. As for Exp. 2, the values measured in the intake and emission of Ca resulted in a lower Ca excreta concentration in the SF-LP group compared to the SF-C group. The significantly higher retention of P in the group of hens fed the SF-LP diets led to a lower emission and excreta concentration of P in this group compared to the results of hens fed the SF-C diet.

## 4. Discussion

The main objective of our study was to investigate the effects of different protein and AA supplies of hens in an SF system on egg production, egg quality traits, apparent ileal AA digestibility, and the retention and emission of N, Ca, and P. The morning diets in previous experiments evaluating the SF system without whole grain feeding contained a higher crude protein content compared to the conventional diets [2,22,23,24]. The investigations of Keshavarz [3,10] showed that egg performance and quality can be maintained without increasing the crude protein content of morning diets. The author did not use a real SF system in his experiments because the AME and Ca contents of the morning and afternoon diets were the same. Based on his study, the crude protein contents of the SF morning and C diets were identical in our Exp. 1 as a new approach in an SF system. Furthermore, the SID concentrations of eight essential AAs were calculated and adjusted in the SF and C diets in Exp. 1. However, the relatively short experimental period in Exp.1 did not allow for an assessment of the long-term effects of SF diets concerning egg production, egg quality, and sustainability traits.

Early studies with free choice feeding have shown that the self-selection of nutrients according to their physiological needs can decrease the daily protein intake of laying hens [2,25]. The SF system offering morning and afternoon diets with different crude protein levels is also applicable to decrease the protein intake of hens as compared to conventional feeding [2,6,8]. Similarly, the SF diets in Exp. 1 decreased the total protein intake of hens compared to the C diet. The lower total protein intake of hens resulted from the decreased protein intake of both the morning and afternoon feeds, like in the study of Keshavarz [3], who investigated a control diet containing 16% crude protein and a morning and an afternoon feed with 16 and 13% crude protein content, respectively. Consistent with our results, some authors demonstrated a decreased FI of laying hens fed SF diets [2,6], while others failed to observe a lower FI with an SF system compared to the conventional feeding [22,26]. However, studies using SF with whole wheat as a morning feed usually presented decreased FI [9,27]. The protein and AA supply in our SF treatment in Exp. 1 proved to be successful because it resulted in similar laying rate, egg weight, and egg mass compared to the C treatment. Furthermore, the lower FI and similar egg performance of hens fed SF diets led to a more advantageous FCR than in the case of hens fed C diets. Previous research has revealed that the SF system is suitable to maintain the same intensity of egg production and egg weight as compared to the conventional feeding not only in laying hens [8,22,26] but in broiler breeders [23,24]. As demonstrated in previous experiments as well [6,9,27], the main advantage of the SF system could be a more favorable FCR, which can provide a compensatory economic benefit during operation for the higher investment cost of two silos for the morning and afternoon feed.

The protein and AA supply of hens in the first type of SF system is based on the theory that the albumen and shell membrane protein synthesis require a higher amount of AAs after ovulation in the morning [4,28]. The presence of an egg in the magnum doubles the rate of protein synthesis compared to another location of the egg in the oviduct [4]. Free choice feeding of a high energy mash, a protein pellet, and oyster shell flakes led to additional protein consumption when an ovum was in the magnum and to a reduction in protein intake with the presence of an egg in the uterus [25]. However, the 16% protein level of the morning feed and 13% protein content of the afternoon feed and the opposite protein concentrations (13% in the morning–16% in the afternoon) resulted in similar egg production, egg weight, FCR, and internal egg quality [3]. Interestingly, the afternoon protein intake of hens was higher in both treatment groups because of the nearly 40–60% FI ration of the morning and afternoon feeds in each treatment group. In a second protein experiment of the current author, the change of 16 and 10% in the crude protein levels in the morning and afternoon feeds again led to similar egg performance results, but in this experiment, the 6% crude protein difference was enough to reach higher protein intake during the morning phase in the 16–10% morning–afternoon protein level group compared to the 10–16% group [10]. In our Exp.1, like in the study of Keshavarz [3], the FIs during the morning and afternoon periods (7 and 9 h, respectively) were nearly 40 and 60% in both the C and SF treatment groups. Hence, the protein intake of hens was higher during the afternoon period not only in the C group but also in the SF group, where the afternoon diet had a lower protein concentration than the morning diet. The hourly protein intakes in the SF group were 0.96 and 1.03 g/hen/hour during the morning and afternoon phases, respectively. The second type of SF system used whole grains with a high energy–low protein content as a morning feed and a high protein–high Ca mixture in the afternoon. The higher protein concentration of the afternoon diet in this SF system had no effect on egg production, egg mass, or FCR compared to the conventional feeding mixture [29]. These observations do not support the hypothesis that adequate protein and AA intake only during the morning is crucial or that acceptable egg production is only possible when the protein and AA intake is higher during the morning phase compared to the afternoon period. The results suggest that optimum performance can be expected if the daily protein and AA intake is adequate, regardless of whether maximum daily protein and AA intake occurs in the morning or in the afternoon period.

In Exp. 2, the LP concept was used for the first time in the SF system. The calculated crude protein level of SF-LP diets was nearly 2% lower than that of SF-C, but the concentrations of the main limiting SID Lys, Met, Met + Cys, Thr, and Val were unchanged. After 6 weeks of short-term experimental feeding, the hens already showed significant responses to the protein-reduced SF diet. The treatments with dietary crude protein contents lower than 15% led to an increased egg laying rate and to a reduced egg weight in the LP group compared to the control SF. As a result of these changes, the daily egg mass did not differ in the two treatment groups. The 3% decrease in the protein content of both the morning and afternoon diets (13 and 13%) decreased egg production by 10% (from 93 to 83%) in comparison to the control treatment (16 and 16%) [3]. However, the egg weight was not significantly different between groups. The author adjusted similar total Lys, Met + Cys, and Trp contents in the diets and did not calculate the digestible essential AA levels. As mentioned before, this experimental setup cannot be characterized as a true SF system, because the dietary energy (AME) and Ca contents of the morning and afternoon feeds were the same. It seems that even nearly twenty years ago, the layer genotype in a conventional system was able to maintain a satisfactory egg production rate with 13–13.5% dietary crude protein levels if the main essential AAs, like Lys, Met, Thr, and Trp [11] or Lys, Met, Trp, Iso, and Val [12] were supplemented to satisfactory levels. The layers of a new genotype from 19 to 32 weeks of age can also produce a similar number of eggs to conventional diets containing 19, 17, 15, and 13% dietary crude protein levels and AA supplementation [15]. However, like in our study, the egg weight was significantly reduced with the decreasing dietary protein level. In our Exp.2, the SID levels of Arg, Leu, Ile, and Trp were not adjusted with crystalline AAs in the formulation of SF-LP diets. However, the SID Arg level of 0.75% in the SF-LP morning diet meets the requirement of the Hy-Line Brown hybrid (Layer 2 phase, 115 g/day/hen FI, Hy-Line Performance Guide, 2024; Hy-Line International) and the ratio of SID Arg to SID Lys (103%) is adequate both in the morning and afternoon SF-LP diets. The concentration of Leu is usually not limited in layer diets containing maize and the recommended dietary 0.81% SID Leu level is fulfilled in each SF-C and SF-LP diet [30]. In contrast to SID Arg and Leu, the dietary levels and intakes of SID Ile and Trp in the SF-LP groups could contribute to the decrease in egg weight compared to the SF-C group. The SID level of Ile (0.60%) and Trp (0.16%) in the SF-C morning feed met the recommendations of the hybrid, and the SID ratios of both AA to SID Lys in the SF-C afternoon feed were appropriate. However, the ratio of SID Ile to SID Lys in both SF-LP diets were 66% instead of the ideal protein reference value of 81% (Hy-Line Performance Guide, 2024; Hy-Line International) and the daily intake of SID Ile was nearly 21% lower in the SF-LP group compared to the SF-C group (505 mg/day/hen vs. 640 mg/day/hen). Similarly, an SID Trp to SID Lys ratio of 18% did not meet the recommended 22% in the SF-LP diets and the daily intake of SID Trp showed an approximately 17% decrease in the SF-LP group compared to the SF-C group (139 g/day/hen vs. 168 g/day/hen). Thus, the essential Ile and Trp could have been limiting AAs in the diets of the SF-LP group in our study. According to our result, the formulation of LP diets with a 2% crude protein reduction in the SF system with already reduced AA levels in the afternoon diets needs a proper adjustment of SID levels of Lys, Met, Met + Cys, Thr, Val, Arg, Leu, Ile, and Trp. Despite the reduced supply of Ile and Trp in the SF-LP group, the egg mass production (g/day) did not change due to the opposite significant changes in egg production % and egg weight. In the study of Peganova and Eder [31] with conventional feeding, maximum daily egg mass was achieved at daily Ile intakes ranging from 412 mg to 770 mg. The reason for this low Ile requirement of maximum egg mass compared with other studies might be the relatively short duration of the trial (8 weeks). In our short 6-week-long study, the intake of Ile was 718 and 566 mg/day/hen in the SF-C and SF-LP groups, respectively, which were in the range observed in the study of the two authors [31]. The authors discussed that a longer experimental period may lead to a reduced egg mass in response to a relatively low Ile intake of Leu less than 500 mg/day/hen. In agreement with this statement, further studies with longer evaluation times are needed to investigate the long-term effects of the SF-LP diets.

Among egg quality traits, only eggshell thickness was significantly affected by dietary treatments in Exp. 1. The different morning and afternoon Ca supply in the SF treatment adjusted to the higher Ca need of the shell calcification significantly improved eggshell thickness in comparison with the C treatment. This increase in eggshell thickness did not result in a significantly higher eggshell strength; however, a higher number of samples could have increased the reliability of the differences between means. These shell quality results were associated with lower Ca intake in the morning and higher Ca intake in the afternoon phase in the SF treatment group compared to the C group. Formation of the eggshell starts in the afternoon and/or early evening, which increases the Ca requirement and absorption of available Ca [32]. Supplying the Ca during the active stages of shell calcification directly from intestinal absorption is more efficient than from bone reserves [33]. The higher amount of absorbed Ca during shell formation in the SF group compared to the C group may explain the shell quality results in our Exp.1. Early studies demonstrated a significant positive effect of the SF system on eggshell thickness and breaking strength [6] and shell deformation [2]. Similarly, a higher eggshell thickness has been observed in hens fed SF diets with a higher Ca content at the time of eggshell formation compared to the conventional diet at 83 weeks of age [34]. However, the SF system did not affect eggshell quality in studies with broiler breeders [23,24]. A lower percentage of soft-shelled eggs was found in the SF treatment group compared to the conventional treatment group of Hy-Line W-36 hens in the late production phase from 60 to 80 weeks of age [26]. The percentage of cracked or damaged eggs in the group of hens fed the SF diets was lower than in the control group from 72 to 83 weeks [34]. Egg quality parameters have not been investigated with hens fed LP diets in SF systems until our Exp. 2. The lower egg weight in the SF-LP group compared to the SF-C group was associated with higher albumen height and Haugh unit. The Haugh unit provides an indication of the freshness of the egg and is calculated with the value of the egg weight and albumen height. It relates to protein synthesis at the time of egg formation, and feeding of SF-LP diets could be related to a better availability of AAs for albumen synthesis [35]. Previous studies with hens in a conventional feeding system also reported that lowering dietary crude protein levels increased the Haugh unit [15,36].

Lowering the crude protein content of diets is one of the most effective techniques to reduce the total nitrogen excretions and ammonia emissions of poultry houses. The reduction in dietary crude protein by 1% can lead to an approximately 10% decrease in N excretion [37]. Free choice feeding of hens with separate energy, protein, and Ca sources decreased the total intake and output of N and increased the N retention significantly [25]. The 16–10% crude protein concentrations in the morning and afternoon SF diets, respectively, decreased total protein/N intake and increased the protein/N retention of hens compared to the control morning–afternoon diets containing 16–16% crude protein contents [10]. Like in our Exp. 1, the N intake of laying hens fed SF diets was shown to be lower than birds fed conventional diets [2]. This lower N intake with similar N retention led to a decrease in the daily N emission of hens by 7% in the SF group compared to the C group in our trial. Most of the ammonia released from poultry manure originates from urinary N (NH_4_^+^-N + uric acid–N) and the breakdown of uric acid is the main source of ammonia [38]. A detailed evaluation of the forms of the emitted N by hens in an SF system has not been provided yet. Our results showed that the SF system not only decreased the total amount of excreted N but that the composition of excreta became more favorable with a lower concentration of NH_4_^+^-N, uric acid–N, and urinary N as compared to the conventional feeding. In Exp. 2, the reduction in dietary crude protein of the morning and afternoon diets in the SF-LP group did not decrease the total N and the measured N forms of excreta in a significant manner compared to the SF-C group. However, the extent of protein reduction applied in this trial resulted in strong tendencies in favor of the SF-LP diets. A low protein layer diet in conventional feeding resulted in a decrease in nitrogen intake and excretion vs. a high protein diet in previous trials [11,39]. Reducing dietary crude protein and maintaining similar essential AA levels in SF diets reduced excreta N and moisture, which is in line with the findings of broiler [40] and layer studies [11] using conventional LP feeding.

Our study investigated the apparent ileal digestibility of AAs in an SF system for the first time. The different composition of morning and afternoon SF diets was able to significantly increase the ileal digestibility of some AAs compared to the conventional feeding in Exp. 1. Among the essential AAs, Lys and Leu responded to SF with significantly increased ileal digestibility. Their increase in digestibility by nearly 3–5% could also contribute to the similar egg performance of SF hens with lower N intake in comparison to hens fed conventional diets. The reason for these results is not clear and needs further investigation. The SF system may decrease the endogenous ratio of some AAs, which can lead higher apparent ileal digestibility values. The afternoon diet can be considered as an LP diet, which may also explain the more favorable ileal digestibility of some AAs. Reduced crude protein feeding generally increases the apparent ileal digestibility of AAs in broilers [41,42] and in growing finishing pigs [43]. The decrease in dietary crude protein level in SF-LP compared to SF-C diets further increased the ileal digestibility of Leu and Ile in experiment 2. The higher digestibility of AAs in LP diets can be explained partly by the larger dietary ratio of crystalline AAs and their better absorption [42]. However, this is true only for Lys in Exp. 1 and does not explain the improved apparent ileal digestibility of other AAs in our study.

In addition to the reduced N emission of layer farms, the SF system may decrease the P excretion of laying hens. In contrast to egg yolk and albumen, the eggshell rich in Ca contains only a small quantity of P [44]. Furthermore, a higher amount of P is needed in the morning to replace bone P that was resorbed during the shell formation of the previous night [45]. Therefore, the relation of P concentrations in the morning and afternoon SF diets is opposite to the relation of the Ca levels. The SF provides the hens with an adequate and higher P level in the morning phase and a low P diet is fed in the afternoon period [8,10,22]. Previous research is limited and showed that SF in organic laying hens resulted in a lower P excretion [46]. In contrast to this result, in Exp. 1, the significantly reduced P intake in the SF group was not associated with significantly changed P excretion and emissions compared to the C group. However, in Exp. 2, the feeding of SF-LP diets led to an increased P retention and decreased P intake and emissions compared to the feeding of SF-C diets. The results so far do not provide a sufficient explanation of how the SF system affects the P balance of laying hens, and the topic needs further investigation.

## 5. Conclusions

In conclusion, the maintenance of high egg production and good egg quality does not require an increased crude protein content in the morning SF feed if the SID levels of important essential AAs in the morning (Lys, Met, Met + Cys, Arg, Thr, Val, Leu, Ile, Trp) and in the afternoon (Lys, Met, Met + Cys) diet meet the conventional requirements. In addition, LP diets with a 2% crude protein reduction in the SF system by maintaining dietary SID concentrations of the main essential AAs (Lys, Met, Met + Cys, Thr, Val) enable a similar feed intake, daily egg mass, and FCR compared to SF diets with normal crude protein levels. However, unadjusted SID levels of other essential AAs, like Ile and Trp in SF-LP diets, may result in a decreased egg weight. The feeding of SF diets with reduced crude protein and adjusted digestible AA content can reduce the N intake and the emissions of the total N and NH_4_^+^-N of laying hens and lead to a more sustainable production than the conventional feeding system. However, further, longer studies are needed to investigate the reliability of our results obtained in short model experiments.

## Figures and Tables

**Table 1:** Composition and nutrient content of experimental diets in Exp. 1 (as fed %).

Ingredients	Control	Split Feeding Diets	
		Morning	Afternoon
Maize	38.70	40.42	39.83
Wheat	20.00	20.00	20.00
Extracted soybean meal	13.01	12.53	6.40
Extracted sunflower meal	8.15	8.00	12.08
Sunflower oil	1.75	1.90	0.94
Wheat flour	2.50	2.99	3.50
Corn DDGS	6.00	6.00	6.00
L-Lysine (Biolys)	0.04	0.10	0.18
DL-Methionine	0.09	0.12	0.10
MCP	0.40	0.38	0.31
Limestone coarse	6.03	4.83	6.92
Limestone fine	2.60	2.00	3.00
Salt	0.23	0.23	0.24
Premix ^1^	0.50	0.50	0.50
Calculated nutrients			
AMEn (MJ/kg)	11.25	11.54	10.86
Crude protein	16.00	16.00	14.72
Crude fat	3.93	4.13	3.14
Crude fiber	4.83	4.85	5.36
Crude ash	12.64	10.84	13.66
SID Lysine	0.63	0.67	0.62
SID Met + Cys	0.60	0.63	0.59
SID Met	0.34	0.37	0.34
SID Arginine	0.90	0.89	0.81
SID Threonine	0.50	0.50	0.45
SID Valine	0.67	0.66	0.61
SID Leucine	1.10	1.10	0.99
SID Isoleucine	0.57	0.57	0.51
SID Tryptophan	0.16	0.16	0.14
Calcium	3.42	2.75	3.88
P total	0.49	0.49	0.48
P available	0.35	0.35	0.33
Analyzed nutrients			
Dry matter	89.18	88.82	89.16
Crude protein	15.63	15.70	14.32
Crude fat	3.95	4.16	3.20
Crude fiber	4.72	4.98	5.10
Calcium	3.68	3.06	4.21
P total	0.46	0.46	0.46

^1^ Premix added to the diets included (per kg of diet) vitamin A (retinyl acetate) 8000 IU, vitamin D_3_ (cholecalciferol) 3000 IU, vitamin E (dl-alpha-tocopherol-acetate) 30 mg, menadione 3.0 mg, thiamine 2.0 mg, riboflavin 6.0 mg, pyridoxin HCl 4.0 mg, cyanocobalamin 0.040 mg, niacin 30 mg, pantothenic acid 8.0 mg, folic acid 1.0 mg, biotin 0.20 mg, choline chloride 147 mg, betaine 180 mg, Zn (as ZnSO_4_H_2_O) 80 mg, Cu (as CuSO_4_5H_2_O) 8 mg, Fe (as FeSO_4_H_2_O) 30 mg, Mn (as MnO) 100 mg, I (as KI) 1.5 mg, Se (as Na_2_SeO_3_) 0.3 mg, AxtraPhy 20,000 TPT 15 mg (300 FTU), Danisco Xilanáz 40,000 TPT 50 mg, Xanthophyll 512 mg, Apoester 8.17 mg, Canthaxanthin 6.8 mg, Carophyll Yellow 87 mg, BHT 25.05 mg.

**Table 2:** Composition and nutrient content of experimental diets in Exp. 2 (as fed %).

Ingredients	Control Diet		Low-Protein Diet	
	Morning	Afternoon	Morning	Afternoon
Maize	32.00	35.00	28.00	30.00
Wheat	33.79	28.31	45.24	40.29
Extracted soybean meal	13.76	13.22	5.15	3.24
Extracted sunflower meal	9.16	7.67	10.00	10.00
Sunflower oil	1.58	1.33	1.32	1.35
L-Lysine (Biolys)	0.13	0.06	0.36	0.32
DL-Methionine	0.11	0.10	0.11	0.14
L-Threonine	0.01	0.00	0.11	0.11
L-Valine	0.00	0.00	0.11	0.12
MCP	0.45	0.36	0.55	0.43
Limestone coarse	4.80	9.00	4.80	9.00
Limestone fine	3.44	4.18	3.44	4.21
Salt	0.28	0.28	0.28	0.28
Premix ^1^	0.50	0.50	0.50	0.50
Calculated nutrients				
AMEn (MJ/kg)	11.27	10.74	11.29	10.82
Crude protein	16.48	15.16	14.40	13.00
Crude fat	3.25	3.00	2.90	2.90
Crude fiber	4.33	3.89	4.29	4.09
Crude ash	12.40	16.94	12.14	16.69
SID Lysine	0.73	0.64	0.73	0.64
SID Met + Cys	0.63	0.59	0.63	0.59
SID Met	0.36	0.34	0.36	0.34
SID Arginine	0.95	0.88	0.75	0.68
SID Threonine	0.53	0.49	0.53	0.49
SID Valine	0.69	0.64	0.69	0.64
SID Leucine	1.14	1.08	0.94	0.86
SID Isoleucine	0.60	0.55	0.48	0.43
SID Tryptophan	0.16	0.14	0.13	0.12
Ca	3.31	5.10	3.31	5.11
P total	0.48	0.43	0.48	0.43
P available	0.33	0.31	0.33	0.31
Analyzed nutrients				
Dry matter	90.15	90.81	90.36	90.72
Crude protein	16.21	15.01	14.53	13.21
Crude fat	3.25	3.04	2.83	3.14
Crude fiber	4.29	4.11	4.37	4.54
Calcium	3.18	4.98	3.16	4.92
P total	0.44	0.38	0.41	0.37

^1^ Premix added to the diets included (per kg of diet) vitamin A (retinyl acetate) 8000 IU, vitamin D_3_ (cholecalciferol) 3000 IU, vitamin E (dl-alpha-tocopherol-acetate) 30 mg, menadione 3.0 mg, thiamine 2.0 mg, riboflavin 6.0 mg, pyridoxin HCl 4.0 mg, cyanocobalamin 0.040 mg, niacin 30 mg, pantothenic acid 8.0 mg, folic acid 1.0 mg, biotin 0.20 mg, choline chloride 147 mg, betaine 180 mg, Zn (as ZnSO_4_H_2_O) 80 mg, Cu (as CuSO_4_5H_2_O) 8 mg, Fe (as FeSO_4_H_2_O) 30 mg, Mn (as MnO) 100 mg, I (as KI) 1.5 mg, Se (as Na_2_SeO_3_) 0.3 mg, AxtraPhy 20,000 TPT 15 mg (300 FTU), Danisco Xilanáz 40,000 TPT 50 mg, Xanthophyll 512 mg, Apoester 8.17 mg, Canthaxanthin 6.8 mg, Carophyll Yellow 87 mg, BHT 25.05 mg.

**Table 3:** Effects of dietary treatments on feed and protein intake of laying hens (mean ± SEM; n = 23 in Exp. 1, n = 24 in Exp. 2).

Experiment	Treatment ^1^	Feed Intake (g/hen/day)			Protein Intake (g/hen/day)		
		Morning	Afternoon	Total	Morning	Afternoon	Total
Experiment 1	C	45.1 ± 0.4	69.0 ± 0.5	114.0 ± 0.9	7.0 ± 0.1	10.8 ± 0.1	17.8 ± 0.1
	SF	42.9 ± 0.4	65.0 ± 0.4	107.8 ± 0.8	6.7 ± 0.1	9.3 ± 0.1	16.0 ± 0.1
	*p* values	<0.001	<0.001	<0.001	<0.001	<0.001	<0.001
Experiment 2	SF-C	44.5 ± 0.3	67.9 ± 0.4	112.5 ± 0.7	7.2 ± 0.1	10.2 ± 0.1	17.4 ± 0.1
	SF-LP	44.6 ± 0.3	67.7 ± 0.5	112.3 ± 0.8	6.5 ± 0.1	8.9 ± 0.1	15.4 ± 0.1
	*p* values	NS ^2^	NS	NS	<0.001	<0.001	<0.001

^1^ C—conventional (control), SF—split feeding; SF-C—split feeding control, SF-LP—split feeding low protein; ^2^ NS—nonsignificant (*p* > 0.05).

**Table 4:** Effects of dietary treatments on calcium and phosphorus intake of laying hens (mean ± SEM; n = 23 in Exp. 1, n = 24 in Exp. 2).

Experiment	Treatment ^1^	Calcium Intake (g/hen/day)			Phosphorus Intake (mg/hen/day)		
		Morning	Afternoon	Total	Morning	Afternoon	Total
Experiment 1	C	1.66 ± 0.01	2.54 ± 0.02	4.20 ± 0.03	207.2 ± 1.6	317.4 ± 2.5	524.8 ± 4.1
	SF	1.31 ± 0.01	2.74 ± 0.02	4.05 ± 0.03	197.3 ± 1.9	298.9 ± 2.0	496.1 ± 3.8
	*p* values	<0.001	<0.001	<0.001	<0.001	<0.001	<0.001
Experiment 2	SF-C	1.42 ± 0.01	3.38 ± 0.02	4.80 ± 0.03	196.0 ± 1.5	258.1 ± 1.5	454.0 ± 2.9
	SF-LP	1.41 ± 0.01	3.33 ± 0.02	4.74 ± 0.03	183.0 ± 1.2	250.4 ± 1.8	433.5 ± 3.0
	*p* values	NS ^2^	NS	NS	<0.001	<0.05	<0.001

^1^ C—conventional (control), SF—split feeding; SF-C—split feeding control, SF-LP—split feeding low protein; ^2^ NS—nonsignificant (*p* > 0.05).

**Table 5:** Performance parameters of laying hens (mean ± SEM; n = 23 in Exp. 1, n = 24 in Exp. 2).

Experiment	Treatment ^1^	Egg Production (%)	Egg Weight (g)	Egg Mass (g/day)	FCR ^2^ (kg/kg)
Experiment 1	C	98.5 ± 0.4	60.1 ± 0.6	59.1 ± 0.6	1.93 ± 0.01
	SF	98.0 ± 0.2	60.3 ± 0.7	59.0 ± 0.7	1.83 ± 0.02
	*p* values	NS ^3^	NS	NS	<0.001
Experiment 2	SF-C	90.78 ± 1.4	63.9 ± 0.7	57.3 ± 0.8	1.97 ± 0.02
	SF-LP	94.75 ± 0.8	59.9 ± 0.7	56.7 ± 0.8	1.99 ± 0.03
	*p* values	<0.05	<0.001	NS ^3^	NS

^1^ C—conventional (control), SF—split feeding; SF-C—split feeding control, SF-LP—split feeding low protein; ^2^ FCR—feed conversion ratio; ^3^ NS—nonsignificant (*p* > 0.05).

**Table 6:** Effect of dietary treatments on the egg quality parameters (mean ± SEM; n = 24).

Parameters	Experiment 1			Experiment 2		
	Treatments ^1^		*p*-Values	Treatments		*p*-Values
	C	SF		SF-C	SF-LP	
Egg weight (g)	59.18 ± 0.87	60.15 ± 0.97	NS ^3^	63.72 ± 0.95	59.85 ± 0.87	<0.05
Eggshell strength (kgf)	5.04 ± 0.29	5.31 ± 0.17	NS	4.04 ± 0.22	4.00 ± 0.20	NS
Eggshell thickness (mm)	0.39 ± 0.07	0.42 ± 0.07	<0.05	0.42 ± 0.01	0.43 ± 0.01	NS
Albumen height (mm)	6.01 ± 0.23	6.49 ± 0.21	NS	6.03 ± 0.34	6.80 ± 0.15	<0.05
Haugh unit	76.31 ± 1.92	79.67 ± 1.61	NS	74.00 ± 2.73	81.74 ± 1.04	<0.05
Yolk color ^2^	14.71 ± 0.11	14.61 ± 0.10	NS	14.54 ± 0.13	14.67 ± 0.10	NS
Yolk height (mm)	15.52 ± 0.28	15.35 ± 0.17	NS	17.44 ± 0.33	17.53 ± 0.23	NS
Yolk diameter (mm)	41.58 ± 0.56	41.47 ± 0.67	NS	40.92 ± 0.47	41.12 ± 0.36	NS
Yolk index	0.37 ± 0.01	0.37 ± 0.01	NS	0.43 ± 0.01	0.43 ± 0.01	NS

^1^ C—conventional (control), SF—split feeding; SF-C—split feeding control, SF-LP—split feeding low protein; ^2^ Yolk color was measured as a DSM Yolk Color Fan number on a scale from 1 to 16; ^3^ NS—nonsignificant (*p* > 0.05).

**Table 7:** Nitrogen intake, retention, and emission of hens (mean ± SEM; n = 23 in Exp. 1, n = 24 in Exp. 2).

Experiment	Treatment ^1^	N Intake (g/day/hen)	N Retention (%)	N Emission (g/day/hen)
Experiment 1	C	2.80 ± 0.02	42.93 ± 1.00	1.60 ± 0.26
	SF	2.51 ± 0.02	40.52 ± 0.80	1.49 ± 0.28
	*p* values	<0.001	NS ^2^	<0.05
Experiment 2	SF-C	2.67 ± 0.02	38.23 ± 1.80	1.65 ± 0.05
	SF-LP	2.42 ± 0.02	35.60 ± 2.16	1.57 ± 0.06
	*p* values	<0.001	NS	NS

^1^ C—conventional (control), SF—split feeding; SF-C—split feeding control, SF-LP—split feeding low protein; ^2^ NS—nonsignificant (*p* > 0.05).

**Table 8:** Dry matter content and concentration of N forms in layer excreta (mean ± SEM; n = 23 in Exp. 1, n = 24 in Exp. 2).

Experiment	Treatment ^2^	Dry Matter (%)	Faecal N	NH_4_^+^-N	Uric Acid–N	Urinary N ^1^	Total N
			mg/g DM				
Experiment 1	C	20.18 ± 0.62	21.73 ± 0.43	7.12 ± 0.10	21.39 ± 0.20	28.51 ± 0.28	50.24 ± 0.54
	SF	21.60 ± 0.41	20.53 ± 0.29	6.63 ± 0.10	20.11 ± 0.19	26.75 ± 0.25	47.28 ± 0.47
	*p* values	NS ^3^	<0.05	<0.001	<0.001	<0.001	<0.001
Experiment 2	SF-C	22.43 ± 0.63	29.97 ± 0.77	6.63 ± 0.18	16.76 ± 0.39	23.39 ± 0.54	53.35 ± 1.26
	SF-LP	25.56 ± 0.51	27.87 ± 0.84	6.38 ± 0.17	16.05 ± 0.34	22.43 ± 0.59	50.30 ± 1.41
	*p* values	*p* < 0.001	NS	NS	NS	NS	NS

^1^ The sum of NH_4_^+^-N and uric acid–N was considered as urinary N content; ^2^ C—conventional (control), SF—split feeding; SF-C—split feeding control, SF-LP—split feeding low protein; ^3^ NS—nonsignificant (*p* > 0.05).

**Table 9:** Effect of dietary treatments on the apparent ileal digestibility of amino acids (%, mean ± SEM; n = 8 in Exp. 1, n = 12 in Exp. 2).

Amino Acids	Experiment 1			Experiment 2		
	Treatments ^1^		*p* Values	Treatments		*p* Values
	C	SF		SF-C	SF-LP	
Ala	79.60 ± 1.21	81.50 ± 1.12	NS ^2^	83.08 ± 0.64	86.71 ± 0.59	<0.001
Arg	82.43 ± 1.00	85.30 ± 1.35	NS	87.08 ± 0.48	86.94 ± 0.74	NS
Asp	74.72 ± 0.99	78.30 ± 1.22	<0.05	79.17 ± 0.71	82.04 ± 0.54	<0.05
Cys	73.83 ± 1.00	75.65 ± 0.82	NS	76.30 ± 0.51	77.73 ± 0.62	NS
Glx	87.06 ± 0.88	87.62 ± 0.72	NS	91.36 ± 0.30	91.22 ± 0.54	NS
Gly	69.73 ± 0.96	74.52 ± 1.26	<0.05	76.27 ± 0.64	76.58 ± 0.58	NS
Ile	79.53 ± 1.41	79.68 ± 1.70	NS	79.66 ± 0.75	83.44 ± 0.57	<0.001
Leu	83.61 ± 0.87	86.87 ± 0.92	<0.05	88.97 ± 0.30	90.36 ± 0.37	<0.05
Lys	73.75 ± 0.85	78.71 ± 0.83	<0.05	79.41 ± 0.83	80.34 ± 0.83	NS
Met	84.54± 1.13	85.07 ± 0.85	NS	88.81 ± 0.60	90.12 ± 0.62	NS
Phe	82.05 ± 0.79	84.40 ± 0.92	NS	86.03 ± 0.58	87.42 ± 0.73	NS
Pro	80.22 ± 0.92	81.74 ± 1.03	NS	84.40 ± 0.75	85.78 ± 0.61	NS
Ser	71.51 ± 1.29	76.04 ± 1.26	<0.05	79.22 ± 0.83	78.92 ± 1.00	NS
Thr	62.08 ± 1.29	64.80 ± 0.95	NS	67.34 ± 1.00	69.44 ± 0.84	NS
Val	75.53 ± 1.04	79.00 ± 1.32	NS	79.92 ± 0.65	80.79 ± 0.64	NS
Total	75.95 ± 0.79	78.31 ± 0.87	NS	81.54 ± 0.34	82.81 ± 0.52	NS

^1^ C—conventional (control), SF—split feeding; SF-C—split feeding control, SF-LP—split feeding low protein; ^2^ NS—nonsignificant (*p* > 0.05).

**Table 10:** The intake, retention, emission, and excreta concentration of calcium (mean ± SEM; n = 23 in Exp. 1, n = 24 in Exp. 2).

Experiment	Treatment ^1^	Calcium			
		Intake (g/day/hen)	Retention (%)	Emission (g/day/hen)	Excreta cc. (mg/g DM)
Experiment 1	C	4.20 ± 0.03	45.39 ± 0.51	2.29 ± 0.03	79.59 ± 0.91
	SF	4.05 ± 0.03	50.13 ± 0.45	2.02 ± 0.02	71.67 ± 1.13
	*p* values	<0.001	<0.001	<0.001	<0.001
Experiment 2	SF-C	4.79 ± 0.03	58.38 ± 0.29	1.99 ± 0.02	67.62 ± 1.19
	SF-LP	4.74 ± 0.03	59.11 ± 0.40	1.94 ± 0.02	63.19 ± 1.30
	*p* values	NS ^2^	NS	NS	<0.05

^1^ C—conventional (control), SF—split feeding; SF-C—split feeding control, SF-LP—split feeding low protein; ^2^ NS—nonsignificant (*p* > 0.05).

**Table 11:** The intake, retention, emission, and excreta concentration of phosphorus (mean ± SEM; n = 23 in Exp. 1, n = 24 in Exp. 2).

Experiment	Treatment ^1^	Phosphorus			
		Intake (g/day/hen)	Retention (%)	Emission (g/day/hen)	Excreta cc. (mg/g DM)
Experiment 1	C	0.52 ± 0.01	26.13 ± 1.00	0.39 ± 0.01	11.98 ± 0.17
	SF	0.50 ± 0.01	23.13 ± 1.92	0.38 ± 0.01	12.62 ± 0.18
	*p* values	<0.001	NS ^2^	NS	<0.05
Experiment 2	SF-C	0.45 ± 0.01	30.90 ± 0.85	0.31 ± 0.01	10.08 ± 0.18
	SF-LP	0.43 ± 0.01	37.87 ± 0.82	0.27 ± 0.01	8.81 ± 0.16
	*p* values	<0.001	<0.001	<0.001	<0.001

^1^ C—conventional (control), SF—split feeding; SF-C—split feeding control, SF-LP—split feeding low protein; ^2^ NS—nonsignificant (*p* > 0.05).

## Data Availability

Data supporting the reported results are contained within the article.
